# Identifying emerging priorities in Knowledge Translation from the perspective of trainees

**DOI:** 10.1186/1472-6963-14-S2-P130

**Published:** 2014-07-07

**Authors:** Kristine Newman, Dwayne Van Eerd, Byron Powell, Robin Urquhart, Evelyn Cornelissen, Vivian Chan, Shalini Lal

**Affiliations:** 1 Daphne Cockwell School of Nursing, Ryerson University, Toronto, Ontario, Canada; 2 Institute for Work & Health, Toronto, Ontario, Canada; 3 Brown School, Washington University in St. Louis, St. Louis, Missouri, USA; 4 Department of Surgery, Dalhousie University, Halifax, Nova Scotia, Canada; 5 Department of Family Practice, University of British Columbia, Vancouver, British Columbia, Canada; 6 Vancouver Coastal Health, Vancouver, British Columbia, Canada; 7 Department of Psychiatry, McGill University, Montreal, Quebec, Canada

## Background

As the Knowledge Translation (KT) field advances, there is an increasing need to identify priorities to help shape future directions for research. An important source of KT priorities is ‘experts’ who are well-established researchers and practitioners. Another potential source for identifying priorities is trainees. Given that many KT trainees are developing their programs of research, understanding their main concerns and priorities for KT research and practice is critical to supporting the development and advancement of KT as a field. The purpose of this study was to identify priorities for research and practice in the KT field from the perspectives of KT researcher and practitioner trainees.

## Materials and methods

The study population was a convenience sample of trainees who are associated with the KT Trainee Collaborative (KTTC) and/or the KT Canada Summer Institutes (KTSI). Trainees affiliated with the KTTC and KTSI were emailed a link to a survey through FluidSurveysTM. Descriptive statistics (e.g., frequency) were calculated for trainee demographics. Open-ended survey responses were analysed using qualitative content analysis; these involved trainees’ priorities for KT research (i.e., topics, methods) and their views on KT topics that do not need further exploration. Trainees’ important KT references for researchers/practitioners were also captured.

## Results

The response rate for the survey was 62% (44/71). Participants were graduate students, post-doctoral fellows, medical residents, and other learners from various disciplines. Ninety-three percent were from Canada and 7% from other countries (USA, UK and Switzerland). Preliminary results suggest research priorities related to: continued KT intervention research, mostly the evaluation of effective KT strategies; a greater focus on novel methodological approaches and innovative strategies; and a better understanding of contextual factors. When asked which topics of KT research/practice do not need further exploration, most indicated the inquiry should be kept broad as KT is a new field; others stated that developing new KT frameworks/models (versus testing/improving current frameworks/models) and simply assessing barriers/facilitators of research use are not advancing the field.

## Conclusions

This study highlights that in addition to ‘experts’ in KT, understanding the main concerns and priorities for KT research and practice from the perspective of trainees is critical to supporting the development and advancement of KT as a field. The findings suggest an emphasis on evaluation of KT interventions, using novel methods for evaluation. The findings of this study, suggest that KT trainees might impact future KT research and practices by identifying KT research priorities.

